# Hepatic Oxidative Stress, Apoptosis, and Inflammation in Broiler Chickens With Wooden Breast Myopathy

**DOI:** 10.3389/fphys.2021.659777

**Published:** 2021-04-14

**Authors:** Tong Xing, Xiaona Pan, Lin Zhang, Feng Gao

**Affiliations:** Key Laboratory of Animal Origin Food Production and Safety Guarantee of Jiangsu Province, Joint International Research Laboratory of Animal Health and Food Safety, Jiangsu Collaborative Innovation Center of Meat Production and Processing, Quality and Safety Control, National Experimental Teaching Demonstration Center of Animal Science, College of Animal Science and Technology, Nanjing Agricultural University, Nanjing, China

**Keywords:** wooden breast, broiler chicken, liver, oxidative stress, apoptosis, inflammation

## Abstract

Wooden breast (WB) syndrome has emerged as a global myopathy in modern commercial broiler chickens, mainly affecting the pectoralis major muscle. Recent evidence suggests that WB myopathy is a systemic disease, which might be accompanied by other physiological disparities and metabolic changes. This study was conducted to systemically investigate the potential physiological changes in liver tissues as well as the possible mechanisms involved to enhance the understanding of the etiology. A total of 93 market-age Arbor Acres male broiler chickens were sampled and categorized into control (CON) and WB groups based on the evaluation of myopathic lesions. Liver samples were collected (*n* = 10 in each group) for histopathological evaluation and biochemical analyses. Results indicated that WB birds exhibited significantly higher plasma aspartate amino transferase, alkaline phosphatase, and gamma glutamyl transpeptidase activities. Histopathological changes in hydropic/fatty degeneration, inflammatory cell infiltration, intrahepatic hemorrhages, elevated myeloperoxidase activity, and overproduction of nitric oxide were observed in WB liver compared with CON, suggesting the occurrence of liver injury in birds affected by WB myopathy. The WB group showed increased levels of reactive oxygen species, oxidative products, as well as enhanced antioxidant capacities in the liver. These changes were associated with impaired mitochondria morphology and mitochondrial dysfunction. WB myopathy also induced mitochondria-mediated hepatic apoptosis by upregulating levels of caspases 3 and 9, altering the expressions of apoptotic B-cell lymphoma-2 family regulators, as well as increasing the release of cytochrome c. The activation of nuclear factor kappa-light-chain-enhancer of activated B cell signaling enhanced the mRNA expression of downstream inflammatory mediators, contributing to the production of inflammatory cytokines in WB liver. Combined, these findings suggest that hepatic disorders may be conjoined with WB myopathy in broiler chickens and indicating systemic physiological disparities, and other metabolic changes accompanying this myopathy need further assessment.

## Introduction

Over the past few decades, the demand for poultry meat has increased notably. Consequently, genetic selection of modern commercial broiler chickens has been pushed toward fast growth and enhanced breast muscle yield (Petracci et al., 2015). Despite achieving extraordinary improvements, this selection pressure accompanied with the modern intense breeding programs have caused the increasing incidence of spontaneous breast muscle abnormalities. These emerging myopathies have drawn worldwide attention due to their high occurrence and negative impacts on meat quality (Petracci et al., 2019). One abnormality, wooden breast (WB) myopathy, is macroscopically characterized by hardened areas, pale ridge-like bulges, as well as occasional appearance of clear viscous fluid, small hemorrhages, and white striping in the pectoralis major (PM) muscle (Sihvo et al., 2014). Due to the unappealing appearance, WB fillets are usually downgraded and used only for highly processed products. Although still usable, WB myopathy seriously impairs the quality and nutritional value of breast meat (Mudalal et al., 2015; Soglia et al., 2016), thereby causing substantial economic losses to the poultry industry.

Extensive studies have been carried out to investigate the histological lesions, physiological properties, and molecular changes involved in the development of WB myopathy. The findings imply that the underlying mechanisms of this growth-related muscular abnormality are complicated and multifaceted processes, which might be related with the abnormal accumulation of endomysial and perimysial connective tissue and the consequent fibrosis, hypoxia, oxidative stress, and inflammatory response as well as metabolic shift (Sihvo et al., 2014; Mutryn et al., 2015; Soglia et al., 2017; Liu et al., 2020). Recently, accumulating evidence suggests that the etiology of WB myopathy is not limited to the PM muscle, but is also associated with perturbations in blood circulation and other organs. Greene et al. (2019) demonstrated that the circulatory oxygen homeostasis was dysregulated in WB myopathic broiler chickens as indicated by the altered pressure of blood gases and hemoglobin levels. Our recent study revealed that the inflammatory cytokines including interleukin (IL)-1β, IL-8, and tumor necrosis factor (TNF)-α were enhanced in the serum of WB affected birds (Xing et al., 2021). Furthermore, lung histopathology of WB myopathic birds exhibited occasional localized multifocal lymphoplasmacytic phlebitis and more foci of chondro-osseous metaplasia compared with the unaffected birds (Lake et al., 2020). WB myopathy increased stress hormone corticosterone levels in plasma and altered expression patterns of stress response-related genes in the liver (Kang et al., 2020). Assessment of potential systemic physiological disparities accompanying WB myopathy might contribute to a profound understanding of its etiology.

The liver is a primary metabolic organ, which has important physiological functions such as biosynthesis, clearance, detoxification, and host defense. Liver damage has become a common disease, which can be caused by various risk factors of xenobiotics, malnutrition, and other chronic diseases (Malhi and Gores, 2008). In patients with Duchenne muscular dystrophy (DMD), liver atrophy was shown to occur concomitantly with skeletal muscle wasting (Moriuchi et al., 1991). Liver abnormalities in mdx dystrophic mice, including decreased glycogen levels and hyperglycemia, have been observed (David et al., 2014). Furthermore, patients with muscular dystrophies showed an increased susceptibility to acute liver failure upon therapeutic paracetamol administration (Pearce and Grant, 2010). It appears that there is a strong link between hepatic disorders and muscular diseases. Interestingly, PM muscle, and liver transcriptome through the ingenuity pathway analysis identified critical transcriptional response network associations in WB myopathic birds (Phillips et al., 2020), suggesting the systemic pathology involved in the progression of this myopathy.

To date, limited data exist on the hepatic changes associated with WB myopathy. The current study was designed to systemically compare the histological and biochemical characteristics and the underlying mechanism causing these differences, if any, in the liver between normal and WB myopathic broiler chickens.

## Materials and Methods

### Experimental Broiler Chickens and Tissue Collection

All experimental procedures and bird managements were approved by the Institutional Animal Care and Use Committee of Nanjing Agricultural University. Broiler chickens used in the current study were all Arbor Acres males raised in three layered cages and received commercially formulated feed and husbandry. Birds were provided *ad libitum* access to feed and water. Birds were vaccinated against Newcastle disease virus, infectious bronchitis virus, and infectious bursal disease virus through neck injection at 11 days of age, using commercially available vaccines. At 42 days of age, a total of 300 live birds were clinically examined for WB myopathy involving visual observations for posture and wing contact as well as bilateral manual palpation for hardness of the pectoralis major (PM) in a cranio-caudal direction by two trained personnel (Papah et al., 2017). This resulted in 63 suspected WB-affected and 30 WB-unaffected broilers. These broilers were electrically stunned (50 V, alternating current, 400 Hz for 5 s each) and exsanguinated via the carotid arteries and jugular veins. Immediately after execution, birds were necropsied, and samples of PM muscle and liver tissues were collected and labeled. Liver tissues from the caudal region of the left lobe were taken and fixed in 4% paraformaldehyde or 2.5% glutaraldehyde for histological evaluation or ultrastructural observation. The remaining liver tissues were frozen in liquid nitrogen and stored at −80°C for biochemical analysis.

### Wooden Breast Myopathy Scoring and Sample Selection

During necropsy, the dissected PM muscle was further evaluated using a more accurate WB myopathy scoring system based on gross lesions and palpable firmness as described by Livingston et al. (2019). Briefly, the ordinal scale ranged from 0 to 3 points, where a score of (0) was used when there was no presence of macroscopic myopathic lesion (normal), (1) was used when the fillets were hard primarily in the cranial region but otherwise pliable (mild), (2) was used when the fillets were hard throughout but flexible in the mid to caudal region (moderate), and (3) was used when the fillets were extremely hard and rigid throughout from cranial region to caudal tip (severe). We found 30 normal, 31 mild, 20 moderate, and 12 severe WB myopathy affected PM muscles among the 93 selected birds. After scoring, 10 liver tissues from birds with normal PM muscle (CON) and 10 samples from birds affected by moderate-to-severe WB myopathy (WB) were randomly selected to evaluate the subsequent biochemical parameters.

### Histopathological Evaluation

For histological analysis, liver tissues were fixed in 4% paraformaldehyde for more than 24 h at room temperature, dehydrated in a graded series of ethanol, trimmed, and embedded in paraffin blocks. Liver sections were cut into 8-μm thickness and mounted on polylysine-coated slides. Subsequently, the slides were rehydrated by a series of incubations in xylene and ethanol solutions and then subjected to hematoxylin and eosin (H&E) and Masson trichrome staining according to the procedures described by Huang et al. (2010) and Xing et al. (2017a). Images were acquired under identical conditions and at the same magnification using a light microscope (Axio Scope.A1, Carl Zeiss, Oberbochen, Germany). Liver tissue was examined for histopathologic changes including the presence of inflammation, intrahepatic hemorrhages, or fibrosis. Assessment of all slides was performed as a blind study to prevent bias in the examination of tissues.

### Determination of Serum Enzymes Activity

Serum enzymatic activities of alanine amino transferase (ALT), aspartate amino transferase (AST), alkaline phosphatase (AKP), and gamma glutamyl transferase (γ-GT) were determined by using the corresponding kits (Nanjing Jiancheng Bioengineering Institute, Nanjing, China) following the manufacturer’s instructions.

### Determination of Myeloperoxidase Activity and Nitric Oxide Level

Myeloperoxidase (MPO) activity and nitric oxide (NO) level were assessed spectrophotometrically with commercial kits purchased from Nanjing Jiancheng Bioengineering Institute (Nanjing, China), per the manufacturer’s instructions. The protein concentration of the liver was determined using a BCA protein assay kit (Pierce Chemical Co., Rockford, IL, United States). The activity of MPO was expressed as units per gram of wet tissue, and the level of NO was expressed as micromoles per gram of protein.

### Analysis of Hepatic Oxidative Products and Antioxidant Ability

Liver tissues were homogenized in 0.9% NaCl buffer and centrifuged at 2,000 × *g* for 10 min at 4°C. The supernatants were collected for the determination of oxidative products and antioxidant ability. Protein concentration was determined using a BCA protein assay kit. The measurements of malondialdehyde (MDA), lipid peroxidation (LPO), and protein carbonyl were performed using corresponding commercial kits obtained from Nanjing Jiancheng Bioengineering Institute (Nanjing, China). The content of the 8-hydroxydeoxyguanosine (8-OHdG) was determined using an ELISA kit obtained from Aogene Bioengineering Institute (Nanjing, China). Results of MDA and protein carbonyl were expressed as micromoles per milligram protein. The content of LPO and 8-OHdG were expressed as moles per milligram protein and nanogram per milligram protein, respectively. The activities of total antioxidant capacity (T-AOC), catalase (CAT), superoxide dismutase (SOD), glutathione peroxidase (GSH-Px), and glutathione S-transferase (GSH-ST) were measured using the corresponding kits (Nanjing Jiancheng Bioengineering Institute, Nanjing, China). The results were all expressed as units per milligram protein.

### Ultrastructural Observation

Liver tissue specimens were fixed in 2.5% glutaraldehyde solution and washed with 0.1 M PBS, followed by postfixing with 1% osmium tetroxide. After washing with PBS, the tissues were hierarchically dehydrated with gradually increasing concentrations of ethanol (30–70%) and then embedded in Spurr’s resin. Samples were embedded in Epon812 and sectioned using an ultra-microtome (RMC Power Tome XL, Leica, Wet-zlar, Germany). Ultrathin sections (30 nm) were collected and stained with 3% uranyl acetate and lead citrate. Ultrastructural changes were examined using a transmission electron microscope (TEM, Hi-tachi H-7650, Tokyo, Japan).

### Determination of Reactive Oxygen Species

Intracellular reactive oxygen species (ROS) in liver was measured using a fluorescent probe, 2,7-dichlorofluorescein diacetate (DCFH-DA, Nanjing Jiancheng Bioengineering Institute, Nanjing, China) as previously described (Xing et al., 2017b). The fluorescence intensity was detected at an excitation wavelength of 500 nm and emission wavelength of 525 nm, respectively, using a fluorescence Microplate Reader (Spectramax M2; Molecular Devices, Sunnyvale, CA, United States).

### Mitochondria Isolation and Mitochondrial Function Assay

The liver mitochondria were extracted as described by Frolova et al. (2019) with some modifications. Briefly, fresh liver tissues were rinsed using phosphate buffer solution (PBS) and minced into mash. Minced tissues were homogenized in chilled isolation buffer (20 mM Tris-HCl, 250 mM sucrose, and 1 mM EDTA, pH 7.4) and then centrifuged at 1,000 × *g* for 15 min at 4°C. The supernatants were collected and centrifuged at 12,000 × *g* for 15 min at 4°C, where mature heavy mitochondria were precipitated. Subsequently, the mitochondria pellets were washed thrice using the isolation buffer. Finally, all mitochondrial fractions were suspended in ice-cold isolation buffer and diluted to a protein concentration of 1 mg/ml. Protein concentration was determined using the BCA protein assay kit. Aliquots of the mitochondrial suspension were stored at −80°C for further use.

Mitochondria membrane potential (Δψm) assay was performed using a JC-1 kit (Solarbio Science & Technology, Co., Ltd, Beijing, China), per the manufacturer’s instructions. Briefly, mitochondrial suspension (20 μl) with a total protein content of 20 μg was mixed with 180 μl of JC-1 dyeing working solution for 10 min, and the fluorescence intensity was measured using a microplate reader. The wavelengths for the detection of the monomeric and aggregated forms of JC-1 were 514/529 and 585/590 nm (excitation/emission).

The mitochondrial swelling was assessed according to Zhang et al. (2015). The obtained liver mitochondria suspension (20 μl) was incubated with 170 μl of swelling assay buffer containing 150 mM KCl, mM HEPES, 2 mM K_2_HPO_4_, 5 mM glutamate, and 5 mM malate to get a 20-μg total protein content. The mitochondrial swelling was triggered by the addition of 10 μl of calcium solution (1 mM). The absorbance was continuously determined at 540 nm for 18 min with an interval of 45 s using a microplate reader. Low mitochondria swelling exhibits high absorbance, and mitochondria with high swelling has low absorbance.

### Apoptotic Nuclei Analysis

The detection of nuclei exhibiting apoptosis was performed using a terminal deoxynucleotidyl transferase (TdT)-mediated dUTP nick-end labeling (TUNEL) kit according to the manufacturer’s instructions (Vazyme Biotech Co., Ltd., Nanjing, China) with minor modifications. Briefly, the paraffin-embedded liver tissues were sectioned at 5 μm, rehydrated by a series of incubations in xylene and ethanol solutions, and permeabilized using proteinase K (20 μg/ml) at 37°C for 25 min. After rinsing in PBS, the sections were incubated with mixed reagents consisting of TdT and dUTP at 37°C for 1 h. The sections were counterstained with 4’,6-diamidino-2-phenylindole (DAPI, Beyotime Biotechnology, Shanghai, China) to label the nuclei. Finally, the TUNEL-positive cells were visualized using a fluorescence microscope (Axio Scope.A1, Carl Zeiss, Oberbochen, Germany). For apoptotic nuclei evaluation, four fields of 137,600 square micrometers per section were randomly selected and analyzed using the Image-Pro Plus software, version 6.0 (Media Cybernetics, Inc., Rockville, MD, United State). The hepatic apoptotic index was calculated as percentage of the total number of nuclei.

### RNA Extraction, cDNA Synthesis, and Quantitative Real-Time PCR

Total RNA was isolated from the liver tissues of broiler chickens using RNAiso Plus reagent (Takara Biotechnology Co., Ltd, Dalian, China) following the manufacturer’s instructions. Total RNA concentration was quantified by measuring the absorbance at 260 nm with a NanoDrop ND-100 spectrophotometer (NanoDrop Technologies, Rockland, DE, United State), and the purity was assessed by determining the ratios of optical density (OD) value at 260 and 280 nm. cDNA was reverse transcribed using a commercial cDNA Synthesis Kit (PrimeScript^TM^ RT Master Mix, Takara) and diluted 20 times with DEPC water before use. Quantitative real-time PCR was performed on an Applied Biosystems 7500 instrument (Foster City, CA, United State) using SYBR Premix EX Taq (Takara, United State). The PCR reaction conditions consisted of denaturation at 95°C for 10 min, followed by 40 cycles of 95°C for 15 s, annealing at 60°C for 1 min, and extension at 60°C for 20 s. Primer sets used for quantitative RT-PCR analysis are listed in Supplementary Table 1. All gene expressions are calculated as the relative fold changes compared with CON, and glyceraldehyde-3-phosphate dehydrogenase (GAPDH) was used as the internal reference to normalize the expression of target genes. Relative mRNA expression was calculated according to the 2^−ΔΔCT^ method.

### Total Protein Extraction and Western Blot Analysis

Frozen liver tissues were homogenized in RIPA lysis buffer (Beyotime Biotechnology, Jiangsu, China) containing 1 mM PMSF. The homogenate was centrifuged at 12,000 × *g* for 20 min at 4°C, and the supernatant was collected. The BCA assay was used to determine protein concentration. Equal amounts of total protein (40 μg) were resolved on 10% SDS-PAGE using a BioRad Electrophoresis System (Richmond, CA, United State) and transferred to a nitrocellulose membrane (Millipore, Merck, Germany). The membranes were blocked with 5% skim milk for 1 h at room temperature and then incubated in primary antibodies against kappa-light-chain-enhancer of activated B cells (NF-κB), inducible nitric oxide synthase (iNOS), cyclooxygenase-2 (COX-2), cytochrome c (Cytc), GAPDH (Servicebio Biological Technology, Wuhan, China), B-cell lymphoma (Bcl)-2 (Boster Biological Technology, Wuhan, China), and caspase3 (Absin Bioscience Inc., Shanghai, China) overnight at 4°C followed by incubation with the corresponding horseradish peroxidase-conjugated secondary antibodies (Bioworld, Nanjing, China) for 1 h. Finally, the membranes were visualized using ECL reagents (Pierce, IL, United States) and scanned using ImageQuant LAS4000 (GE, CT, United State). The density of each band was quantified by using Quantity One software (Bio-rad) and normalized to its respective housekeeping protein (GAPDH). All protein contents are calculated as the relative fold changes compared with CON.

### Statistical Analysis

Data were analyzed by one-way analysis of variance (ANOVA) using SAS 9.12 (2003; SAS Inst. Inc., Cary, NC), and the differences between individuals were compared using Student’s t-tests. Data were reported as means ± SE. Significance was considered when *P* ≤ 0.05, and a trend was indicated when 0.05 < *P* < 0.1.

## Results

### Enzymatic Activities in the Serum of Wooden Breast Myopathic Birds

Measuring levels of serum ALT, AST, AKP, and other enzymes provides a clinical sign of liver injury and ascertains the severity of liver disease. As exhibited in Table 1, the activities of AST, AKP, and γ-GT were elevated by 73.3 ± 15.3% (*P* < 0.01), 63.7 ± 10.6% (*P* < 0.01), and 46.1 ± 3.0% (*P* < 0.01), respectively, in the serum of WB affected broiler chickens compared with CON. No significant difference in ALT activity was observed between the two groups (*P* > 0.05).

**TABLE 1:** Enzymatic activities in serum of broiler chickens with normal (CON) and wooden breast (WB) pectoralis major muscle (*n* = 10).

Items^1^	Category		SEM	*P* value
	CON	WB		
ALT activity (U/L)	1.74	1.76	0.12	ns
AST activity (U/L)	21.07^b^	36.52^a^	2.44	***
AKP activity (U/L)	455.45^b^	745.77^a^	39.25	***
γ-GT activity (U/L)	24.50^b^	35.81^a^	0.62	***

^*a,b*^Mean values within the same row followed by different superscript letters indicating significance (*P* < 0.05). ***Significant difference at *P* < 0.001. ns, indicates no significant difference. ^1^SEM, standard error of mean; ALT, alanine amino transferase; AST, aspartate amino transferase; AKP, alkaline phosphatase; γ-GT, gamma glutamyl transpeptidase.

### Histopathological Observation and Biochemical Parameters in the Liver of Wooden Breast Myopathic Birds

**FIGURE 1:**
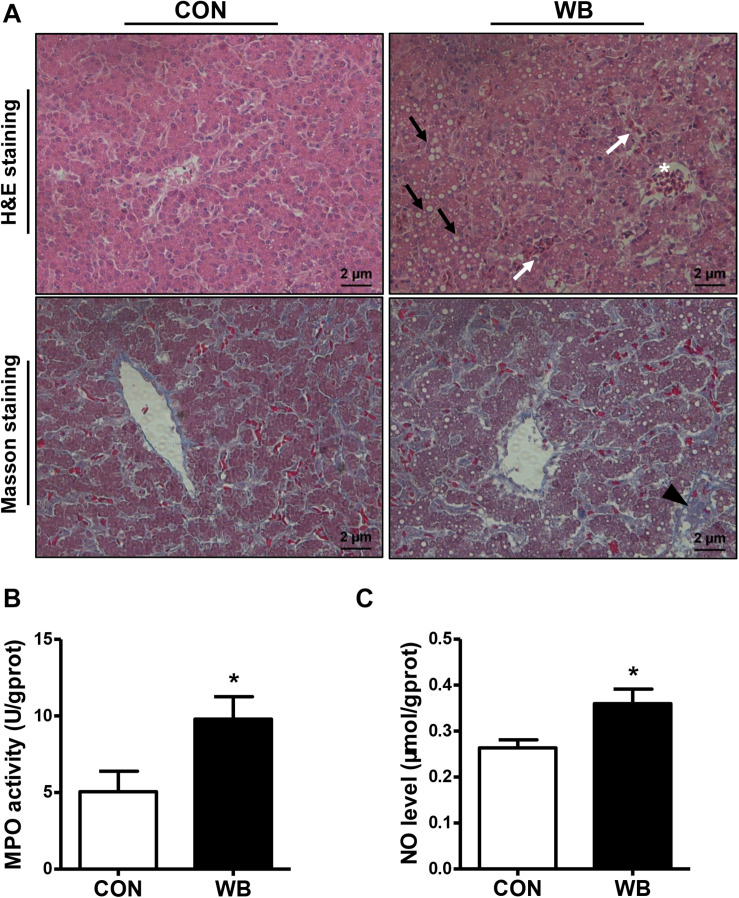
Histopathological observation and biochemical parameters in liver tissues of broiler chickens with normal (CON) and wooden breast (WB) pectoralis major muscle. **(A)** Representative images of hematoxylin and eosin (H&E) and Masson staining of liver, indicating the widespread lesions with hydropic/fatty degeneration (black arrowhead), inflammatory cell infiltration (*), and intrahepatic hemorrhages (white arrowhead) as well as occasional fibrosis (black triangle) in WB. **(B)** Myeloperoxidase (MPO) activity and (**C**) nitric oxide (NO) level. Data are expressed as the mean ± SE (*n* = 10). *P < 0.05.

We stained the liver tissues using H&E and Masson staining to reveal the damage caused by WB myopathy (Figure 1A). Histology of the liver tissues from the CON group showed normal structures with regular morphology. On the contrary, the liver tissue of WB broiler chickens showed widespread lesions with hydropic/fatty degeneration (indicated by black arrows), infiltration of inflammatory cells (indicated by ^∗^), and severe intrahepatic hemorrhages (indicated by white arrows). In addition, occasional collagen deposition or fibrosis (indicated by black triangle) were simultaneously observed in the liver of WB birds. Both MPO activity and NO production were significantly elevated in WB compared with CON (*P* < 0.05; Figures 1B,C).

### Hepatic Oxidative Products and Antioxidant Ability of Wooden Breast Myopathic Birds

As exhibited in Table 2, WB myopathy induced oxidative stress and led to damage in the cellular biomacromolecules in the liver tissues as indicated by the considerable elevated (*P* < 0.05) contents of MDA, LPO, protein carbonyl, and 8-OHdG. Additionally, the liver samples from WB broiler chickens exhibited significantly higher (*P* < 0.05) activities of T-AOC, CAT, SOD, GSH-Px, and GSH-ST than those from the CON birds. These results implied a disturbed redox status in the liver tissue of WB myopathic birds.

**TABLE 2:** Oxidative products and antioxidant ability in liver tissues of broiler chickens with normal (CON) and wooden breast (WB) pectoralis major muscle (*n* = 10).

Items^1^	Category		SEM	*P* value
	CON	WB		
MDA (nmol/mg protein)	0.99^b^	1.16^a^	0.06	*
LPO (mol/mg protein)	0.33^b^	0.42^a^	0.03	*
Protein carbonyl (nmol/mg protein)	1.44^b^	1.70^a^	0.08	*
8-OHdG (ng/g protein)	0.41^b^	0.51^a^	0.06	*
T-AOC (U/mg protein)	3.67^b^	4.45^a^	0.15	**
CAT (U/mg protein)	32.60^b^	47.01^a^	2.56	**
SOD (U/mg protein)	186.13^b^	211.32^a^	6.98	*
GSH-Px (U/mg protein)	25.81^b^	31.21^a^	1.55	*
GSH-ST (U/mg protein)	14.86^b^	23.53^a^	1.40	***

^*a,b*^Mean values within the same row followed by different superscript letters indicating significance (*P* < 0.05). *P < 0.05. **Significant difference at *P* < 0.01. ***significant difference at *P* < 0.001. ^1^SEM, standard error of mean; MDA, malondialdehyde; LPO, lipid peroxidation; 8-OHdG, 8-hydroxydeoxyguanosine; T-AOC, total antioxidant capacity; CAT, catalase; SOD, superoxide dismutase; GSH-Px, glutathione peroxidase; GSH-ST, glutathione S-transferase.

### Wooden Breast Myopathy Induced Liver Mitochondria Morphology Changes and Mitochondrial Dysfunction

**FIGURE 2:**
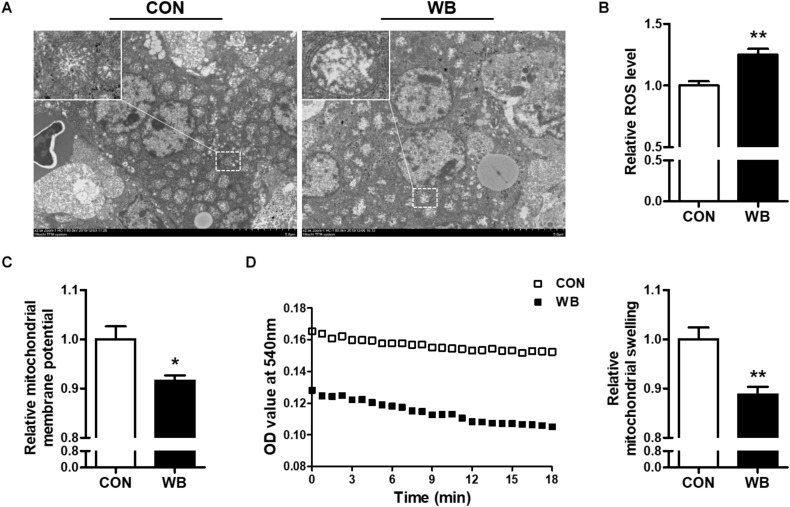
Mitochondria morphology and mitochondrial function changes in the liver tissues of broiler chickens with normal (CON) and wooden breast (WB) pectoralis major muscle. **(A)** Representative transmission electron microscope images of liver. **(B)** Relative reactive oxygen species (ROS) level. **(C)** Relative mitochondrial membrane potential. **(D)** Relative mitochondrial swelling. Data are expressed as the mean ± SE (*n* = 10). **P < 0.01 and *P < 0.05.

The ultrastructure of the mitochondria of liver tissues from the CON group was well developed with intact membrane integrity and rich cristae density, whereas the collapse of cristae and membrane swelling was observed in the liver tissues of the WB group (Figure 2A). The production of ROS was significantly increased in WB when compared with CON (*P* < 0.01; Figure 2B). We further detected Δψm and mitochondrial swelling to evaluate mitochondrial function changes. Results indicated that Δψm presented a significant decrease in the WB group compared with the CON group (*P* < 0.05). Consistently, the mitochondria in the WB birds had significantly decreased OD values at 540 nm triggered by calcium compared with those of the CON birds (*P* < 0.01), showing that the mitochondria in the liver tissue of birds affected by WB myopathy were prone to swelling.

### Wooden Breast Myopathy Induced Apoptosis in Chicken Liver

**FIGURE 3:**
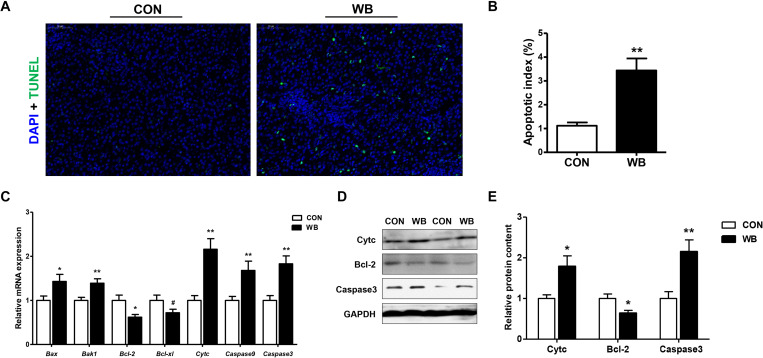
Apoptotic status and apoptosis-related mediators in the liver tissues of broiler chickens with normal (CON) and wooden breast (WB) pectoralis major muscle. **(A)** Representative images of terminal deoxynucleotidyl transferase-mediated dUTP nick-end labeling (TUNEL) staining of liver. **(B)** Relative percentage of hepatocyte apoptosis. **(C)** Relative mRNA expression of Bcl-2-associated X protein (Bax), Bcl-2 antagonist or killer 1 (Bak1), B cell lymphoma (Bcl)-2, Bcl-xl, cytochrome c (Cytc), caspase 9, and caspase3. **(D)** Representative Western blotting images of Cytc, Bcl-2, and caspase 3. **(E)** Relative protein content of Cytc, Bcl-2, and caspase 3. Data are expressed as the mean ± SE (*n* = 10). **P < 0.01, *P < 0.05, and ^#^0.05 < *P* < 0.1.

Apoptotic hepatocytes were detected using TUNEL staining as exhibited in Figure 3A. WB myopathy significantly increased apoptotic index of hepatocyte when compared with the CON group (*P* < 0.01; Figure 3B). The mRNA expressions of pro-apoptotic factors including Bcl-2-associated X protein (*Bax*, *P* < 0.05), Bcl-2 antagonist or killer 1 (*Bak1*, *P* < 0.01), and *Cyt c* (*P* < 0.01) were upregulated, whereas the antiapoptotic regulators of *B cell lymphoma (Bcl)-2* (*P* < 0.05) and *Bcl-xl* (*P* < 0.1) were downregulated in the WB group compared with the CON group (Figure 3C). In addition, the transcription of caspase 9 and caspase 3 were significantly enhanced in WB compared with CON (*P* < 0.01). Consistently, WB myopathy increased the protein contents of Cytc and caspase 3, but decreased the Bcl-2 protein content in comparison with the CON group (*P* < 0.05; Figures 3D,E).

### Wooden Breast Myopathy Induced Inflammatory Responses in Chicken Liver

**FIGURE 4:**
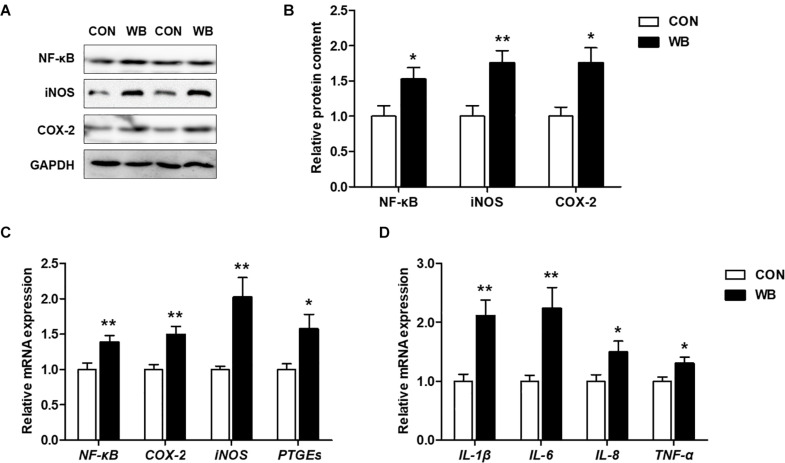
Inflammatory mediators and proinflammatory cytokines in the liver tissues of broiler chickens with normal (CON) and wooden breast (WB) pectoralis major muscle. **(A)** Representative Western blotting images of nuclear factor kappa-light-chain-enhancer of activated B cells (NF-κB), inducible nitric oxide synthase (iNOS), and cyclooxygenase-2 (COX-2). **(B)** Relative protein content of NF-κB, iNOS, and COX-2. **(C)** Relative mRNA expression of NF-κB, iNOS, COX-2, and prostaglandin E synthetases (PTGEs). **(D)** Relative mRNA expression of pro-inflammatory cytokines including interleukin (IL)-1β, IL-6, IL-8, and tumor necrosis factor (TNF)-α. Data are expressed as the mean ± SE (*n* = 10). **P < 0.01 and *P < 0.05.

The protein contents of NF-κB, iNOS, and COX-2 were significantly increased in the liver of WB birds compared with the CON birds (*P* < 0.05, Figures 4A,B). Similarly, WB myopathy enhanced the transcription of *NF-*κ*B*, *iNOS*, *COX-2*, and prostaglandin E synthetases (*PTGEs*) in chicken liver in comparison with the CON group (*P* < 0.05, Figure 4C). Furthermore, WB broiler chickens exhibited significantly increased mRNA expressions of pro-inflammatory cytokines including IL-1β, IL-6, IL-8, and TNF-α in the liver compared with the CON birds (*P* < 0.05, Figure 4D). These results suggested that WB myopathy induced inflammatory responses in the liver by activating inflammatory mediators and enhancing production of proinflammatory cytokines.

## Discussion

WB myopathy has been identified as an emerging muscular disease in modern broiler chickens over the past decade. Chronic WB myopathy-affected muscles exhibit histological lesions of polyphasic myodegeneration, myofiber necrosis, small regenerating myofibers, inflammatory cell infiltration, as well as deposition of lipid and connective tissue (Sihvo et al., 2014). Growing evidence suggests that the excessive development of breast muscle leads to the hypertrophy of myofiber, thereby reducing perimysial and endomysial space available for capillaries and compromising blood supply (Velleman, 2019), which possibly trigger an intricate pathogenesis including oxidative stress, impaired calcium homeostasis, inflammatory responses, and metabolic shifts (Petracci et al., 2019). Meanwhile, the aberrant accumulation of interstitial fibrotic tissues and a consequent increase in the spaces between muscle fibers might be associated with a lower capillary density and a greater intercapillary distance, which could further deteriorate the microvascular architecture and aggravate this situation (Soglia et al., 2017). Besides breast muscle abnormalities, broilers afflicted with WB myopathy also exhibit secondary pathophysiological perturbations in blood circulation and in other organ systems (Lake et al., 2020; Phillips et al., 2020).

The liver is defined as the primary internal organ for poultry exerting a variety of metabolic and homeostatic functions including digestion, metabolism, biosynthesis, excretion, and detoxification. The disruption of hepatic function has been implicated to reduce growth performance and threaten the health of birds, causing economic losses to the poultry industry (Zaefarian et al., 2019). In addition, mammalian studies indicate that liver disease might be implicated as a conjoint pathological mechanism underlying several metabolic disorders including neuromuscular disease (Pearce and Grant, 2010; David et al., 2014). Recent studies have likewise assessed the alterations of transcriptome and stress response genes in both liver and muscle of WB myopathic birds and revealed that the etiology of this myopathy is not limited to muscle, but is a systemic pathology (Kang et al., 2020; Phillips et al., 2020). Therefore, this study was conducted to investigate the histological and biochemical status and to depict the possible mechanistic changes involved in the liver of myopathic birds for better understanding of the molecular basis of the underlying pathological process and thereby promoting the healthy production of broiler chickens.

Injury to the liver tissue can lead to the release of various hepatic enzymes into the bloodstream. Increases in plasma levels of aminotransferases, AKP, and γ-GT are widely used as diagnostic markers of hepatic damage (Li et al., 2019). Herein, we reported that serum AST, AKP, and γ-GT activities were significantly elevated in WB birds, suggesting that liver injury occurs in this myopathy. Similarly, broilers affected by other myopathies such as white striping (WS) and dorsal cranial myopathy exhibited increased levels of ALT and AST in the serum (Kuttappan et al., 2013; Sesterhenn et al., 2017). However, Kuttappan et al. (2013) observed the unchanged γ-GT and decreased AKP activity in the serum of WS-affected birds compared with the normal. We ascribed this inconsistency to the different stages of myopathy progression. Since WS and WB are both growth-associated myopathies sharing similar histological lesions (Sihvo et al., 2014), the myopathic aberrations of PM muscle might start with the onset of WS and progress into WB during the whole growth period (Griffin et al., 2018). Consistent with the elevations of indicators of hepatic damage in the plasma, we observed widespread lesions with hydropic/fatty degeneration, infiltration of inflammatory cells, occasional fibrosis, and severe intrahepatic hemorrhages in liver sections of WB myopathic birds. The increase in MPO activity and the overproduction of NO further implied the infiltration of neutrophil and mononuclear cells, and confirmed the induction of the inflammatory process in the liver tissue (Aktan, 2004; Loria et al., 2008).

Oxidative stress occurs when the balance of pro-oxidants and endogenous antioxidants in a living system is disturbed, which can lead to the overproduction of free radicals (Sies et al., 2017). Previous studies suggested the presence of altered redox homeostasis and oxidative stress as possible biological processes linked with the pathogenesis of WB disease (Abasht et al., 2016; Papah et al., 2018). In addition, the excessive formation of ROS was directly observed in WS and WB-affected PM muscle (Salles et al., 2019; Pan et al., 2020). Intracellular macromolecules are vulnerable to free radicals, and the resultant lipid peroxidation, modification of proteins, and nucleic acid breaks may further contribute to structural collapse and dysfunction. Herein, we observed an aberrant ROS accumulation and augmented levels of MDA, LPO, protein carbonyl, and 8-OHdG, indicating that oxidative stress occurs in the liver of WB-affected birds. Oxidative stress or disturbed redox state has been implicated as a crucial mediator contributing to hepatic damage and the progression of pathological liver disorders (Zhu et al., 2012). Aflatoxin B1 administration induced oxidative stress in the liver of broilers, which contributed to hepatic dysfunction characterized by pallor discoloration, enlargement, and necrosis (Li et al., 2019). Intraperitoneal injection of hydrogen peroxide triggered hepatic oxidative stress by increasing ROS level and contents of oxidative products, thereby exerting a negative impact on the histomorphology and redox status in the liver, as well as the resultant decline in growth performance of broilers (Chen et al., 2018). Furthermore, oxidative markers could serve as prognostic indicators of liver damage and chronic hepatic diseases such as non-alcoholic steatohepatitis and liver fibro-proliferative disease (Cichoż-Lach and Michalak, 2014). To counteract oxidative stress, organisms generally stimulate multiple layers of antioxidant defense system to reduce the formation of excessive free radicals. As expected, we observed significantly enhanced activities of T-AOC, CAT, SOD, GSH-Px, and GSH-ST in the liver of the WB group compared with the CON group. Accordingly, the antioxidant enzyme defensive system was activated in the PM muscle of WB myopathic broilers (Pan et al., 2020). The inhibited activities of GSH-Px and GSH-ST were also observed in the PM muscle of severe WS-affected birds (Salles et al., 2019), suggesting that the activation of antioxidant armamentarium depends on the stage and severity of injury or disease. The mitochondria not only constitute primary sources of ROS but also are vulnerable to oxidative attack due to the high content of phospholipid and protein in their membranes (Cadenas and Davies, 2000). In the current study, the ultrastructural examination indicated the damaged mitochondrial structure in WB liver. In addition, the WB liver mitochondria exhibited loss of Δψm and were prone to go through swelling, indicating an impaired function. In the meantime, mitochondria dysfunction may interact with cellular redox environment, contributing to ROS overproduction and the impaired antioxidant defense system (Balaban et al., 2005). Collectively, these results demonstrated the occurrence of oxidative stress in the liver of WB myopathic birds as evidenced by the disturbed redox homeostasis and mitochondria damage, which possibly contribute to hepatic pathological changes.

Oxidative stress triggered by various insults or pathological states is closely associated with the induction of programmed cell apoptosis (Ryter et al., 2007). The present study revealed a significantly increased number of hepatocytes undergoing apoptosis in WB demonstrated by the TUNEL assay. The apoptosis process is executed through the caspase family, among which, caspase3 plays a vital role in mediating both intrinsic and extrinsic signaling pathways. The present results indicated the mRNA expression of *caspase 3* and *9* as well as the protein content of caspase 3 that was upregulated in the WB liver compared with the CON. Similarly, the occurrence of apoptosis is involved in liver injury, alcoholic and non-alcoholic steatohepatitis, and chronic liver disease (Aizawa et al., 2020). The mitochondrial and endoplasmic reticulum-mediated intrinsic apoptosis is usually triggered by a variety of stimuli such as calcium overload, ROS overproduction, and unfolded protein response. In addition, loss of Δψm is an important hallmark in apoptosis and occurs in the early phase of mitochondria-mediated apoptosis (Kinnally et al., 2011). Based on the elevated ROS accumulation and mitochondrial dysfunction in WB liver, we speculated that the activation of apoptosis was mitochondria mediated caspase dependent. The mitochondrial pathway is regulated by pro- and antiapoptotic Bcl-2 family members. The imbalance of these regulators causes the permeation of the mitochondrial outer membrane and promotes the release of pro-apoptotic protein, thereby activating the caspase cascade (Xing et al., 2019). Therefore, the increased levels of Bax and Bak1, decreased levels of Bcl-2 and Bcl-xl, as well as the release of Cytc confirmed the activated mitochondrial pathway were involved in liver apoptosis of WB myopathic birds.

Hepatocytes undergoing injury can promote the release of cytokines and recruit inflammatory cells, such as neutrophils and macrophages to clean up debris and stimulate regeneration (Malhi and Gores, 2008). In this study, we observed the upregulated mRNA expression of proinflammatory cytokines including *IL-1*β, *IL-6*, *TNF-*α, and *IL-8* in the liver of WB compared with CON. TNF-α acts as a potent activator of both proinflammatory and proapoptotic pathways, exerting important roles in the pathogenesis of liver injury (Schwabe and Brenner, 2006). IL-1β might contribute to the pathogenesis of liver damage as IL-1β knockout mice showed attenuated hepatocellular damage, steatosis, and fibrosis in atherogenic diet-induced steatohepatitis (Kamari et al., 2011). Therefore, these dysregulated cytokines could further lead to immune disorder and contribute to the aggravation of liver damage. This result also supports our recent finding of the systemic inflammatory response in WB myopathic broilers as implied by the elevation of circulating cytokines (Xing et al., 2021). Besides the secretion of cytokines, inflammatory cells can also produce excessive oxygen free radicals to attack host cells, leading to hepatocyte damage (Zhu et al., 2012). NF-κB is a central regulator in mediating liver inflammatory responses by controlling the expression of cytokines; the activation of NF-κB signaling has been implicated in various liver diseases (Luedde and Schwabe, 2011). In accordance with these studies, the expression of NF-κB was enhanced, and the expressions of its downstream targets including iNOS, COX-2, and PTGEs were upregulated in WB compared with those in CON. Meanwhile, these increased inflammatory mediators could further contribute to the exacerbation of inflammatory progression and cytokine production (Aktan, 2004; Subbaramaiah et al., 2012).

## Conclusion

In summary, this study provides evidence of liver damage in birds affected by WB myopathy primarily by impaired liver morphology as well as elevated serum AST, AKP, and γ-GT activities. Oxidative stress in WB liver triggered by the excessive ROS accumulation might be associated with disturbance of redox status and mitochondrial dysfunction. Additionally, the present study confirms the mitochondria-mediated hepatocyte apoptosis and NF-κB signaling-regulated inflammatory response, which possibly contribute to the aggravation of liver injury of WB myopathic birds. In general, our results strongly suggest that hepatic disorders might be strongly correlated with WB myopathy and provide evidence to explain the possible mechanisms involved in these perturbations. Further studies are needed to assess systemic physiological disparities and other metabolic changes accompanying this myopathy for further recognition of its etiology.

## Data Availability Statement

The original contributions presented in the study are included in the article/Supplementary Material, further inquiries can be directed to the corresponding author.

## Ethics Statement

The animal study was reviewed and approved by Institutional Animal Care and Use Committee of Nanjing Agricultural University.

## Author Contributions

TX and FG conceived and designed the study. TX, XP, and LZ performed the experiments and conducted the data analysis. TX drafted the manuscript. All authors contributed to the article and approved the submitted version.

## Conflict of Interest

The authors declare that the research was conducted in the absence of any commercial or financial relationships that could be construed as a potential conflict of interest.

## References

[B1] Abasht B. Mutryn M. F. Michalek R. D. Lee W. R. (2016). Oxidative stress and metabolic perturbations in wooden breast disorder in chickens. *PLoS One* 11:e0153750. 10.1371/journal.pone.0153750 27097013 PMC4838225

[B2] Aizawa S. Brar G. Tsukamoto H. (2020). Cell death and liver disease. *Gut Liver* 14 20–29. 10.5009/gnl18486 30917630 PMC6974333

[B3] Aktan F. (2004). iNOS-mediated nitric oxide production and its regulation. *Life Sci.* 75 639–653. 10.1016/j.lfs.2003.10.042 15172174

[B4] Balaban R. S. Nemoto S. Finkel T. (2005). Mitochondria, oxidants, and aging. *Cell* 120 483–495. 10.1016/j.cell.2005.02.001 15734681

[B5] Cadenas E. Davies K. J. (2000). Mitochondrial free radical generation, oxidative stress, and aging. *Free Radical Bio. Med.* 29 222–230. 10.1016/S0891-5849(00)00317-8 11035250

[B6] Chen X. Gu R. Zhang L. Li J. Jiang Y. Zhou G. (2018). Induction of nuclear factor-κB signal-mediated apoptosis and autophagy by reactive oxygen species is associated with hydrogen peroxide-impaired growth performance of broilers. *Animal* 12 2561–2570. 10.1017/S1751731118000903 29720292

[B7] Cichoż-Lach H. Michalak A. (2014). Oxidative stress as a crucial factor in liver diseases. *World J. Gastroenterol.* 20 8082–8091. 10.3748/wjg.v20.i25.8082 25009380 PMC4081679

[B8] David I. Lau X. Flores M. Trieu J. Gehrig S. M. Chee A. (2014). Dysfunctional muscle and liver glycogen metabolism in mdx dystrophic mice. *PLoS One* 9:e91514. 10.1371/journal.pone.0091514 24626262 PMC3953428

[B9] Frolova M. S. Marchenkov V. V. Vekshin N. L. (2019). Disruption of flavin homeostasis in isolated rat liver mitochondria. *Biochem. Bioph. Res. Commun.* 516 1211–1215. 10.1016/j.bbrc.2019.07.021 31300198

[B10] Greene E. Flees J. Dadgar S. Mallmann B. Orlowski S. Dhamad A. (2019). Quantum blue reduces the severity of woody breast myopathy via modulation of oxygen homeostasis-related genes in broiler chickens. *Front. Physiol.* 10:1251. 10.3389/fphys.2019.01251 31632293 PMC6781743

[B11] Griffin J. R. Moraes L. Wick M. Lilburn M. S. (2018). Onset of white striping and progression into wooden breast as defined by myopathic changes underlying Pectoralis major growth. Estimation of growth parameters as predictors for stage of myopathy progression. *Avian Pathol.* 47 2–13. 10.1080/03079457.2017.1356908 28714747

[B12] Huang Y. Yan X. Zhu M. J. Mccormick R. J. Ford S. P. Nathanielsz P. W. (2010). Enhanced transforming growth factor-β signaling and fibrogenesis in ovine fetal skeletal muscle of obese dams at late gestation. *Am. J. Physiol. Endoc. Metab.* 298 E1254–E1260. 10.1152/ajpendo.00015.2010 20371734 PMC2886526

[B13] Kamari Y. Shaish A. Vax E. Shemesh S. Kandel-Kfir M. Arbel Y. (2011). Lack of interleukin-1α or interleukin-1β inhibits transformation of steatosis to steatohepatitis and liver fibrosis in hypercholesterolemic mice. *J. Hepatol.* 55 1086–1094. 10.1016/j.jhep.2011.01.048 21354232 PMC3210940

[B14] Kang S. W. Kidd M. T. Kadhim H. J. Shouse S. Kong B. C. (2020). Characterization of stress response involved in chicken myopathy. *Gen. Comp. Endocr.* 295:113526. 10.1016/j.ygcen.2020.113526 32540490

[B15] Kinnally K. W. Peixoto P. M. Ryu S.-Y. Dejean L. M. (2011). Is mPTP the gatekeeper for necrosis, apoptosis, or both? *BBA Mol. Cell Res.* 1813 616–622. 10.1016/j.bbamcr.2010.09.013 20888866 PMC3050112

[B16] Kuttappan V. A. Huff G. R. Huff W. E. Hargis B. M. Apple J. K. Coon C. (2013). Comparison of hematologic and serologic profiles of broiler birds with normal and severe degrees of white striping in breast fillets. *Poult. Sci.* 92 339–345. 10.3382/ps.2012-02647 23300298

[B17] Lake J. A. Brannick E. M. Papah M. B. Lousenberg C. Abasht B. (2020). Blood gas disturbances and disproportionate body weight distribution in broilers with wooden breast. *Front. Physiol.* 11:304. 10.3389/fphys.2020.00304 32317988 PMC7154160

[B18] Li S. Muhammad I. Yu H. Sun X. Zhang X. (2019). Detection of Aflatoxin adducts as potential markers and the role of curcumin in alleviating AFB1-induced liver damage in chickens. *Ecotox. Environ. Saf.* 176 137–145. 10.1016/j.ecoenv.2019.03.089 30925330

[B19] Liu J. Puolanne E. Schwartzkopf M. Arner A. (2020). Altered sarcomeric structure and function in woody breast myopathy of avian pectoralis major muscle. *Front. Physiol.* 11:287. 10.3389/fphys.2020.00287 32328000 PMC7160512

[B20] Livingston M. L. Landon C. Barnes H. J. Brake J. (2019). White striping and wooden breast myopathies of broiler breast muscle is affected by time-limited feeding, genetic background, and egg storage. *Poult. Sci.* 98 217–226. 10.3382/ps/pey333 30101277

[B21] Loria V. Dato I. Graziani F. Biasucci L. M. (2008). Myeloperoxidase: a new biomarker of inflammation in ischemic heart disease and acute coronary syndromes. *Mediators Inflamm.* 2008:135625. 10.1155/2008/135625 18382609 PMC2276594

[B22] Luedde T. Schwabe R. F. (2011). NF-κB in the liver—linking injury, fibrosis and hepatocellular carcinoma. *Nat. Rev. Gastro. Hepat.* 8 108–118. 10.1038/nrgastro.2010.213 21293511 PMC3295539

[B23] Malhi H. Gores G. J. (2008). Cellular and molecular mechanisms of liver injury. *Gastroenterology* 134 1641–1654. 10.1053/j.gastro.2008.03.002 18471544 PMC2553363

[B24] Moriuchi T. Fujii Y. Kagawa N. Hizawa K. (1991). Autopsy study on the weight of the heart, liver, kidney and brain in Duchenne muscular dystrophy. *Tokushima J. Exp. Med.* 38 5–13. 1949000

[B25] Mudalal S. Lorenzi M. Soglia F. Cavani C. Petracci M. (2015). Implications of white striping and wooden breast abnormalities on quality traits of raw and marinated chicken meat. *Animal* 9 728–734. 10.1017/S175173111400295X 25500004

[B26] Mutryn M. F. Brannick E. M. Fu W. Lee W. R. Abasht B. (2015). Characterization of a novel chicken muscle disorder through differential gene expression and pathway analysis using RNA-sequencing. *BMC Genomics* 16:399. 10.1186/s12864-015-1623-0 25994290 PMC4438523

[B27] Pan X. Zhang L. Xing T. Li J. Gao F. (2020). The impaired redox status and activated Nrf2/ARE pathway in wooden breast myopathy in broiler chickens. *Asian. Austral. J. Anim.* 34 652–661. 10.5713/ajas.19.0953 32299158 PMC7961296

[B28] Papah M. B. Brannick E. M. Schmidt C. J. Abasht B. (2017). Evidence and role of phlebitis and lipid infiltration in the onset and pathogenesis of Wooden Breast Disease in modern broiler chickens. *Avian Pathol.* 46 623–643. 10.1080/03079457.2017.1339346 28609139

[B29] Papah M. B. Brannick E. M. Schmidt C. J. Abasht B. (2018). Gene expression profiling of the early pathogenesis of wooden breast disease in commercial broiler chickens using RNA-sequencing. *PLoS One* 13:e0207346. 10.1371/journal.pone.0207346 30517117 PMC6281187

[B30] Pearce B. Grant I. S. (2010). Acute liver failure following therapeutic paracetamol administration in patients with muscular dystrophies. *Anaesthesia* 63 89–91. 10.1111/j.1365-2044.2007.05340.x 18086077

[B31] Petracci M. Mudalal S. Soglia F. Cavani C. (2015). Meat quality in fast-growing broiler chickens. *World. Poultry Sci. J.* 71 363–374. 10.1017/S0043933915000367

[B32] Petracci M. Soglia F. Madruga M. Carvalho L. Ida E. Estévez M. (2019). Wooden−breast, white striping, and spaghetti meat: causes, consequences and consumer perception of emerging broiler meat abnormalities. *Compr. Rev. Food Sci. Foog* 18 565–583. 10.1111/1541-4337.12431 33336940

[B33] Phillips C. A. Reading B. J. Livingston M. Livingston K. A. Ashwell C. M. (2020). Evaluation via supervised machine learning of the broiler pectoralis major and liver transcriptome in association with the muscle myopathy wooden breast. *Front. Physiol.* 11:101. 10.3389/fphys.2020.00101 32158398 PMC7052112

[B34] Ryter S. W. Kim H. P. Hoetzel A. Park J. W. Nakahira K. Wang X. (2007). Mechanisms of cell death in oxidative stress. *Antioxid. Redox Sign.* 9 49–89. 10.1089/ars.2007.9.49 17115887

[B35] Salles G. B. C. Boiago M. M. Silva A. D. Morsch V. M. Gris A. Mendes R. E. (2019). Lipid peroxidation and protein oxidation in broiler breast fillets with white striping myopathy. *J. Food Biochem.* 43:e12792. 10.1111/jfbc.12792 31353592

[B36] Schwabe R. F. Brenner D. A. (2006). Mechanisms of liver injury. I. TNF-α-induced liver injury: role of IKK, JNK, and ROS pathways. *Am. J. Physiol. Gastr. Liver Physiol.* 290 G583–G589. 10.1152/ajpgi.00422.2005 16537970

[B37] Sesterhenn R. Siqueira F. Hamerski A. Driemeier D. Valle S. Vieira S. (2017). Histomorphometric study of the anterior latissimus dorsi muscle and evaluation of enzymatic markers of broilers affected with dorsal cranial myopathy. *Poult. Sci.* 96 4217–4223. 10.3382/ps/pex252 29053816

[B38] Sies H. Berndt C. Jones D. P. (2017). Oxidative stress. *Annu. Rev. Biochem.* 86 715–748. 10.1146/annurev-biochem-061516-045037 28441057

[B39] Sihvo H. K. Immonen K. Puolanne E. (2014). Myodegeneration with fibrosis and regeneration in the pectoralis major muscle of broilers. *Vet. Pathol.* 51 619–623. 10.1177/0300985813497488 23892375

[B40] Soglia F. Gao J. Mazzoni M. Puolanne E. Cavani C. Petracci M. (2017). Superficial and deep changes of histology, texture and particle size distribution in broiler wooden breast muscle during refrigerated storage. *Poult. Sci.* 96 3465–3472. 10.3382/ps/pex115 28595272

[B41] Soglia F. Mudalal S. Babini E. Di Nunzio M. Mazzoni M. Sirri F. (2016). Histology, composition, and quality traits of chicken Pectoralis major muscle affected by wooden breast abnormality. *Poult. Sci.* 95 651–659. 10.3382/ps/pev353 26706363

[B42] Subbaramaiah K. Morris P. G. Zhou X. K. Morrow M. Du B. Giri D. (2012). Increased levels of COX-2 and prostaglandin E2 contribute to elevated aromatase expression in inflamed breast tissue of obese women. *Cancer Discov.* 2 356–365. 10.1158/2159-8290.CD-11-0241 22576212 PMC3398487

[B43] Velleman S. G. (2019). Recent developments in breast muscle myopathies associated with growth in poultry. *Annu. Rev. Anim. Biosci.* 7 289–308. 10.1146/annurev-animal-020518-115311 30256651

[B44] Xing T. Gao F. Tume R. K. Zhou G. Xu X. (2019). Stress effects on meat quality: a mechanistic perspective. *Compr. Rev. Food Sci. Food* 18 380–401. 10.1111/1541-4337.12417 33336942

[B45] Xing T. Luo D. Zhao X. Xu X. Gao F. (2021). Enhanced cytokine expression and upregulation of inflammatory signaling pathways in broiler chickens affected by wooden breast myopathy. *J. Sci. Food Agr.* 101 279–286. 10.1002/jsfa.10641 32623748

[B46] Xing T. Wang C. Zhao X. Dai C. Zhou G. Xu X. (2017a). Proteome analysis using isobaric tags for relative and absolute analysis quantitation (iTRAQ) reveals alterations in stress-induced dysfunctional chicken muscle. *J. Agr. Food Chem.* 65 2913–2922. 10.1021/acs.jafc.6b05835 28304171

[B47] Xing T. Zhao X. Wang P. Chen H. Xu X. Zhou G. (2017b). Different oxidative status and expression of calcium channel components in stress-induced dysfunctional chicken muscle. *J. Anim. Sci.* 95 1565–1573. 10.2527/jas.2016.0868 28464077

[B48] Zaefarian F. Abdollahi M. R. Cowieson A. Ravindran V. (2019). Avian liver: the forgotten organ. *Animals* 9:63. 10.3390/ani9020063 30781411 PMC6406855

[B49] Zhang J. Hu Z. P. Lu C. Yang M. X. Zhang L. L. Wang T. (2015). Dietary curcumin supplementation protects against heat-stress-impaired growth performance of broilers possibly through a mitochondrial pathway. *J. Anim. Sci.* 93 1656–1665. 10.2527/jas.2014-8244 26020187

[B50] Zhu R. Wang Y. Zhang L. Guo Q. (2012). Oxidative stress and liver disease. *Hepatol. Res.* 42 741–749. 10.1111/j.1872-034X.2012.00996.x 22489668

