# Supplementary material for: Hepatic Oxidative Stress, Apoptosis, and Inflammation in Broiler Chickens With Wooden Breast Myopathy
Source: Front Physiol. 2021 Apr 14;12:659777. doi: 10.3389/fphys.2021.659777 (PMC8081064; doi:10.3389/fphys.2021.659777)
Supplement: Supplementary file 1 [file Table_1.DOCX]

Supplementary Material

# Supplementary Table 1. Primer sets used for quantitative RT-PCR analysis.

| Gene name | Accession no. | Product size | Direction | Sequence (5'→3') |
| --- | --- | --- | --- | --- |
| *GAPDH* | NM_204305.1 | 144bp | Forward | ACATGGCATCCAAGGAGTGAG |
|  |  |  | Reverse | GGGGAGACAGAAGGGAACAGA |
| *Bak-1* | NM_001030920.1 | 272bp | Forward | ACCCGGAGATCATGGAGA |
|  |  |  | Reverse | GATGCCTTGCTGGTAGACG |
| *CytC* | NM_001079478.1 | 138bp | Forward | TGTCCAGAAATGTTCCCAGTGC |
|  |  |  | Reverse | CCTTTGTTCTTATTGGCATCTGTG |
| *Bcl-2* | NM_205339.2 | 205bp | Forward | TGTTTCTCAAACCAGACACCAA |
|  |  |  | Reverse | CAGTAGGCACCTGTGAGATCG |
| *Bcl-xl* | NM_001025304.1 | 190bp | Forward | CTTTCAGCGACCTCACCTC |
|  |  |  | Reverse | ACAATGCGTCCCACCAGT |
| *Bax* | XM_015290060.2 | 108bp | Forward | GGTGACAGGGATCGTCACAG |
|  |  |  | Reverse | TAGGCCAGGAACAGGGTGAA |
| *Caspase3* | NM_204725.1 | 139bp | Forward | TGGCCCTCTTGAACTGAAAG |
|  |  |  | Reverse | TCCACTGTCTGCTTCAATACC |
| *Caspase9* | XM_424580.6 | 241bp | Forward | GAAGGGAGCAAGCACGAC |
|  |  |  | Reverse | GGTTGGACTGGGATGGAC |
| *NFκB* | NM_205134.1 | 162bp | Forward | TCAACGCAGGACCTAAAGACAT |
|  |  |  | Reverse | GCAGATAGCCAAGTTCAGGATG |
| *iNOS* | NM_204961.1 | 82bp | Forward | CCTGGAGGTCCTGGAAGAGT |
|  |  |  | Reverse | CCTGGGTTTCAGAAGTGGC |
| *COX-2* | NM_001167719.1 | 84bp | Forward | TGTCCTTTCACTGCTTTCCAT |
|  |  |  | Reverse | TTCCATTGCTGTGTTTGAGGT |
| *PTGEs* | NM_001194983.1 | 115bp | Forward | GTTCCTGTCATTCGCCTTCTAC |
|  |  |  | Reverse | CGCATCCTCTGGGTTAGCA |
| *IL-6* | NM_204628.1 | 106bp | Forward | AAATCCCTCCTCGCCAATCT |
|  |  |  | Reverse | CCCTCACGGTCTTCTCCATAAA |
| *TNF-α* | NM_204267.1 | 71bp | Forward | GCCCTTCCTGTAACCAGATG |
|  |  |  | Reverse | ACACGACAGCCAAGTCAACG |
| *Il-1β* | NM_204524.1 | 86bp | Forward | CAGCAGCCTCAGCGAAGAG |
|  |  |  | Reverse | CTGTGGTGTGCTCAGAATCCA |
| *IL-8* | NM_205498.1 | 199bp | Forward | GGCTTGCTAGGGGAAATGA |
|  |  |  | Reverse | AGCTGACTCTGACTAGGAAACTGT |
